# Functional assessment in patients with castration-resistant prostate cancer treated with darolutamide: results from the DaroAcT study

**DOI:** 10.1093/oncolo/oyae287

**Published:** 2024-10-25

**Authors:** Tomasz M Beer, Daniel J George, Neal D Shore, Kerri Winters-Stone, Jeffrey S Wefel, Frank Verholen, Shankar Srinivasan, Jorge Ortiz, Alicia K Morgans

**Affiliations:** Knight Cancer Institute, Oregon Health and Science University, Portland, OR 98239, United States; Duke Cancer Institute, Duke University Shool of Medicine, Durham, NC, United States; Carolina Urologic Research Center, Myrtle Beach, SC, United States; Knight Cancer Institute, Oregon Health and Science University, Portland, OR 98239, United States; Division of Oncological Sciences, School of Medicine, Oregon Health and Science University, Portland, OR, United States; Division of Cancer Medicine, The University of Texas MD Anderson Cancer Center, Houston, TX, United States; Bayer Consumer Care, Basel, Switzerland; Bayer Healthcare Pharmaceuticals, Inc., Whippany, NJ, United States; Bayer Healthcare Pharmaceuticals, Inc., Whippany, NJ, United States; Dana-Farber Cancer Institute, Boston, MA, United States

**Keywords:** adverse effects, darolutamide, gait analysis, physical functional performance, prostate cancer

## Abstract

**Background:**

Androgen receptor inhibitors (ARIs) are approved for the treatment of advanced prostate cancer; however, some patients may experience symptoms and side effects that hinder their physical functioning. The Timed Up and Go (TUG) and Short Physical Performance Battery (SPPB) tests are used to assess physical functioning in older adults and are recommended assessments for patients with prostate cancer, despite lacking validation in this setting.

**Methods:**

DaroAct (NCT04157088) was an open-label, multicenter, phase 2b study designed to evaluate the effects of the ARI darolutamide (lead-in phase) and darolutamide vs enzalutamide (randomized phase) on physical functioning in men with castration-resistant prostate cancer (CRPC). Only the lead-in phase, in which participants received darolutamide 600 mg twice daily, was completed. The TUG and SPPB tests were used to assess physical functioning.

**Results:**

The lead-in phase enrolled 30 participants. During 24 weeks of treatment, 8 (32.0%) of 25 evaluable participants exhibited clinically meaningful worsening in TUG from baseline (primary endpoint). At the week 24 visit, 5 (21.7%) of 23 participants had worsening in TUG time, and 8 (33.3%) of 24 participants had worsening in SPPB score. Because only 48% of participants had the same outcome on the TUG and SPPB tests, the study was terminated without initiating the randomized comparison.

**Conclusion:**

Most participants showed no clinically meaningful worsening in physical functioning after 24 weeks of darolutamide treatment, but poor agreement between tests was observed. Tools to accurately and consistently measure the impact of ARIs on physical functioning in patients with CRPC are needed.

## Teaser

DaroAcT was a phase 2b study designed to evaluate the effects of treatment with darolutamide versus enzalutamide on physical functioning in patients with castration-resistant prostate cancer using the TUG and SPPB tests. Results of the lead-in phase are reported.

**Implications for practice**

Androgen receptor inhibitors are approved to treat patients with advanced prostate cancer but are associated with symptoms and side effects that may hinder physical functioning. In the DaroAct study of patients with castration-resistant prostate cancer, most participants treated with darolutamide (an androgen receptor antagonist) showed no clinically meaningful worsening in physical functioning. However, there was poor agreement of results between the 2 tests used to measure physical functioning (Timed Up and Go test and Short Physical Performance Battery test). These results highlight the need to identify the best tool(s) that accurately assess the impact of treatments on physical functioning in patients with advanced prostate cancer.

## Introduction

Androgen deprivation therapy (ADT) is the backbone of systemic therapy for advanced prostate cancer,^1,2^ and novel hormonal therapies (androgen receptor inhibitors [ARIs]), such as darolutamide, enzalutamide, or apalutamide, are among the most frequently used life-prolonging therapies.^3^ However, these hormonal therapies are associated with adverse events, including effects on patients’ physical and cognitive functioning. The frequency and severity of these effects may vary between ARIs, potentially explained by preclinical evidence that suggests differences in blood-brain barrier penetration and related effects on the central nervous pathways and receptors, such as gamma-aminobutyric acid (GABA) receptors.^4,5^ These interactions may lead to impaired cognitive function, increased risk of falls, fatigue, dizziness, and serious neurologic adverse events, such as seizures or convulsions.^6^ In the large pivotal, randomized, phase 3 trials of darolutamide, enzalutamide, and apalutamide in patients with nonmetastatic castration-resistant prostate cancer (nmCRPC), falls and fractures were among the more frequently observed adverse events.^7-9^ ADT therapies in general are often associated with a loss of lean body mass resulting in global muscle weakness (sarcopenia), an increase deposition of subcutaneous adipose tissue, and overall weight gain, further contributing to the deterioration of physical function.^10,11^ In one prospective single-center study, enzalutamide in combination with ADT demonstrated significant declines in functional capacity as measured by cardiopulmonary exercise testing, which was partially mitigated by supervised exercise, although this approach has limited scalability.^12^ Few other studies have investigated the potential effects of novel ARIs specifically on patients’ physical functioning, although detrimental effects in some patients with advanced prostate cancer have been noted.^13,14^

Physical functioning in patients with prostate cancer has primarily been assessed by 2 common functional tests, the Timed Up and Go (TUG) test and the Short Physical Performance Battery (SPPB).^15-22^ The TUG and SPPB tests are highly recommended by the Oncology EDGE Task Force on Prostate Cancer Outcomes for measuring physical functioning^23^; although these tests have yet to be validated in the prostate cancer setting, they have been validated in older adult populations.

The TUG test is a simple and reliable method for assessing lower extremity mobility and the risk of falls.^24-26^ The test measures how long it takes for a person to stand up from a chair, walk 3 meters (~10 feet; typically at a comfortable pace and with their usual walking aids, as necessary), turn around, walk back to the chair, and sit down again.^27,28^ A faster time indicates better physical functioning.^29^ The TUG test is often used and recommended as a screening test for the risk of falls in inpatient and community settings,^24-26,29^ and also to determine frailty status among older adults.^28,30^ Although the TUG test is recommended for the assessment of physical functioning specifically in patients with prostate cancer, only a few studies have published results of the TUG test in this population. One study showed that patients with nonmetastatic prostate cancer who were starting ADT had a gradual decline in TUG time over 36 months that was not seen in the control group^31^; however, 2 other studies reported stable TUG times in patients with prostate cancer who were starting ADT^32^ or already receiving ADT.^33^ Additional studies have evaluated the impact of exercise programs on physical functioning in patients with prostate cancer, again with mixed results.^18,19,34^

The SPPB is an assessment tool developed by the National Institute on Aging for evaluating lower extremity mobility and functioning in older adults.^35,36^ The battery consists of a balance test, assessing static and dynamic balance; a gait speed test, assessing usual walking pace; and a chair stand test, assessing lower extremity strength.^36^ The SPPB has been frequently used to assess risk of falls in community-dwelling older adults^37,38^ and is recommended by guidelines for risk assessment, alongside the TUG test.^39^ Notably, poor performance in the SPPB is associated with an increased risk of all-cause mortality, even after adjusting for potential confounding factors, including age, sex, body mass index, and cardiovascular/metabolic comorbidities.^40^ As with the TUG test, few studies have published results of the SPPB test specifically in patients with prostate cancer, with conflicting findings. One study found that SPPB scores were abnormally low among older patients with prostate cancer who were receiving ADT and worsened over time in approximately 20% of patients.^16^ In contrast, another study reported comparable SPPB scores between patients with nonmetastatic prostate cancer who were starting ADT vs a control group not receiving ADT.^20^ A third study similarly reported comparable SPPB scores between patients with biochemical recurrence of prostate cancer already receiving ADT vs those with a history of prostate cancer not receiving ADT.^17^ Mixed results were also reported among studies using the SPPB test to evaluate the impact of exercise programs on physical functioning.^21,22,41^

Darolutamide is an androgen receptor antagonist whose distinct structure has been shown to result in lower blood-brain barrier penetration than enzalutamide and apalutamide in a preclinical xenograft model, with brain-plasma drug ratios of 1.9%-3.9% for darolutamide compared with 27% and 62% for enzalutamide and apalutamide, respectively.^4^ Thus, darolutamide offers a biologically plausible potential for less frequent and severe mobility-related (eg, fractures and falls) and central nervous system–related adverse events than apalutamide and enzalutamide.^4,7-9,42,43^ The overall efficacy and tolerability profile of darolutamide has been shown in several clinical trials in patients with nmCRPC,^7,44^ metastatic CRPC (mCRPC),^45-47^ and metastatic hormone-sensitive prostate cancer (mHSPC)^48,49^; however, further evaluation of the impact of darolutamide treatment on the frequency and severity of mobility- and central nervous system–associated adverse events and overall physical functioning is needed, and no randomized comparisons of central nervous system–associated adverse events between different ARIs have been reported.

DaroAcT (ClinicalTrials.gov Identifier NCT04157088) was a phase 2b study designed to evaluate the effects of treatment with darolutamide versus enzalutamide on physical functioning in patients with CRPC using the TUG and SPPB tests. DaroAcT was terminated prior to the start of the randomized phase, since the results of the lead-in phase did not fulfill the prespecified protocol criteria of ≥85% concordance of individual participant results between the TUG and SPPB tests; thus, we present the results of the lead-in phase herein.

## Methods

### Study design

DaroAcT was designed as a randomized, open-label, multicenter, phase 2b study comparing darolutamide with enzalutamide in participants with CRPC who had not been previously treated with darolutamide, enzalutamide, or apalutamide. The study was designed to have 2 phases (Figure S1). The first was a lead-in phase to assess study feasibility, including compliance and variability associated with the TUG and SPPB assessments. In the lead-in phase, 30 participants were to be treated with darolutamide 600 mg (2 × 300 mg tablets) twice daily, a dose based on the phase 3 ARAMIS study in patients with nmCRPC.^7^ Primary analysis of the lead-in phase was to occur when at least 30 participants had been in the study for ≥24 weeks, unless participants discontinued due to withdrawal, death, or were lost to follow-up. The second planned phase, to begin after primary evaluation of findings from the lead-in phase, was to randomize 120 additional participants in a 1:1 ratio to receive either darolutamide 600 mg twice daily or enzalutamide 160 mg once daily. Participants were to receive treatment until toxicity or disease progression, and the study was to continue until ≥52 weeks after the initial dose of study treatment was administered to the last participant unless the participant discontinued the study due to death, withdrawal, or being lost to follow-up. After the end of study treatment, further therapy for each participant was at the discretion of the investigator. All participants were required to have CRPC and to continue to receive ADT of the investigator’s choice (luteinizing hormone-releasing hormone [LHRH] agonist/antagonists) as standard therapy or had to have had an orchiectomy. The randomized phase of the study was to begin only after data from the lead-in phase enabled the study steering committee to evaluate the feasibility of proceeding; no other interim analyses were planned.

The study protocol was reviewed and approved by the ethics committee at each participating site, and the study was conducted in accordance with the principles of the Declaration of Helsinki and the International Council for Harmonisation guideline regarding Good Clinical Practice. All participants provided written informed consent.

### Participants

Eligible participants were adults aged ≥18 years who had histologically or cytologically confirmed nmCRPC or mCRPC. The study defined CRPC by disease progression despite ADT that may have presented as either a confirmed rise in serum prostate-specific antigen (PSA) levels, as defined by the Prostate Cancer Working Group 3, the progression of pre-existing disease, and/or the appearance of new metastases. Participants were also required to have a Karnofsky Performance Status of ≥80% at screening and a life expectancy of ≥1 year.

Participants were excluded from the study if they had received prior treatment with a second-generation ARI, such as enzalutamide, apalutamide, or darolutamide, or any investigational ARI, or had disease that progressed while receiving abiraterone acetate and discontinued within 6 months before enrollment. Participants were also excluded if they had visceral metastasis, known metastatic brain or meningeal tumors, or any prior malignancy except adequately treated basal cell or squamous cell carcinoma of the skin, superficial bladder cancer that had not spread behind the connective tissue layer, or any other cancer for which treatment had been completed ≥3 years before the start of the study and from which the patient was disease free. Participants with mCRPC were excluded if they had received chemotherapy or >2 prior lines of systemic anticancer therapy, excluding treatment with an LHRH agonist/antagonist or orchiectomy. Participants were also ineligible if they had any clinically significant cognitive or physical functioning limitations.

### Study endpoints and assessments

The study’s primary endpoint was the proportion of participants with a clinically meaningful worsening in TUG at any time during the 24 weeks from baseline (inclusive of assessments at weeks 12 and 24), defined as a slowing of ≥1 second in TUG time from baseline.^23^ Key secondary endpoints included the proportion of participants with a worsening in TUG time at week 12, at week 24, and at week 52, and the proportion of participants with a clinically meaningful worsening in SPPB total score at week 12, at week 24, and at week 52, with worsening in SPPB score defined as a decline of ≥0.5 points in SPPB total score.^23^ The thresholds for clinically meaningful worsening of TUG time and SPPB score were based on guidance from the Oncology EDGE Task Force on Prostate Cancer Outcomes for measuring physical functioning.^23^ These endpoints were also assessed at the end of the lead-in phase at intervals of every 3 months. To proceed forward from the lead-in phase to the randomized phase, the following criteria were to be met: ≤20% patients present worsening in TUG time, ≤20% patients present worsening of SPPB score, and ≥85% of individual participant results for the TUG and SPPB tests showing the same outcome.

For both the TUG and SPPB tests, all site staff and those identified or assigned as test assessors completed training to standardize the assessments. Video and printed training materials were also made available throughout the study for research staff to reference. The TUG test was performed as described previously, with trained research staff measuring the time required for a patient to rise from an armchair, walk 3 meters, turn around, walk back to the chair, and sit down again.^27^ The 3 timed tests of the SPPB were administered in the following order: balance test, usual gait speed test, and chair stand test.^35,36^ The SPPB summary scores were obtained on a 12-point scale, with each individual test scoring up to 4 points and a greater score indicating a better result (eg, 0 [worst result] to 12 [best result]).^35,36^

Safety of study treatment was documented as treatment-emergent adverse events (TEAEs), serious TEAEs, and TEAEs leading to discontinuation. Adverse events of special interest (fractures, falls, and hypothyroidism) were also reported. Adverse events were defined using the Medical Dictionary for Regulatory Activities (MedDRA), with severity graded according to National Cancer Institute Common Terminology Criteria for Adverse Events (NCI-CTCAE) Version 5.0. Physical examinations, clinical laboratory values, and vital signs were also assessed throughout the study.

### Statistical analysis

There was no formal statistical analysis for the lead-in phase of the study. Continuous data were summarized using descriptive statistics (mean, standard deviation), while categorical data were presented as frequency counts, percentages, and 95% confidence intervals (CIs). At each assessment, the status of TUG test time was summarized as the categories of improved, stable, or worsened. The percentage of participants with a decline of ≥0.5 points in SPPB total score was summarized.

## Results

### Participants

Thirty participants were enrolled in the lead-in phase of the study at 7 centers across the United States and received darolutamide between December 17, 2019, and July 8, 2022. Participants had a median age of 75.5 years (53.3% were aged ≥75 years), 46.7% were White, and 43.3% had nmCRPC (Table 1). The majority (*n* = 21 [70.0%]) of participants were still receiving study treatment when the study was terminated at the end of the lead-in phase, ending on-study treatment. Additional reasons for discontinuation of study treatment (at any time during the study) included physician decision (*n* = 4 [13.3%]), participant withdrawal (*n* = 4 [13.3%]), and death (*n* = 1 [3.3%]).

**Table 1.** Baseline demographics and clinical characteristics.

Characteristics	Darolutamide 600 mg twice daily (*N* = 30)
Age	
Median (range), years	75.5 (55-92)
<75 years, *n* (%)	14 (46.7)
≥75 years, *n* (%)	16 (53.3)
Race, *n* (%)	
White	14 (46.7)
Black or African American	7 (23.3)
Not reported	9 (30.0)
Ethnicity, *n* (%)	
Not Hispanic or Latino	26 (86.7)
Hispanic or Latino	1 (3.3)
Not reported	3 (10.0)
Karnofsky performance scale, *n* (%)	
100	15 (50.0)
90	9 (30.0)
80	6 (20.0)
Metastatic disease at study entry, *n* (%)	
No metastases	13 (43.3)
Bone metastases with/without lymph node metastases	15 (50.0)
Visceral metastases with/without lymph node or bone metastases	1 (3.3)
Nonregional lymph node metastases only	1 (3.3)
Time from diagnosis, mean (SD), months	123.4 (81.4)
Type of prior systemic anticancer therapy, *n* (%)	
Chemotherapy^a^	2 (6.7)
LHRH agonist	19 (63.3)
LHRH antagonist	2 (6.7)
Received prior radiotherapy, *n* (%)	16 (53.3)
Type of prior radiotherapy, *n* (%)	
External beam radiotherapy	11 (36.7)
High dose rate brachytherapy	1 (3.3)
Image-guided radiation therapy	2 (6.7)
Intensity-modulated radiation therapy	6 (20.0)
Stereotactic body radiation therapy	1 (3.3)
Other	1 (3.3)
Received prior surgery, *n* (%)	18 (60.0)
Type of prior surgery, *n* (%)	
Prostatectomy	7 (23.3)
Radical prostatectomy	5 (16.7)
Transurethral resection prostate	4 (13.3)
Pelvic lymph node dissection	1 (3.3)
Other	4 (13.3)

^a^Prior chemotherapy was permitted only among patients with nonmetastatic castration-resistant prostate cancer.

Abbreviaiton: LHRH, luteinizing hormone-releasing hormone.

### Physical functioning endpoints and study termination

In the primary endpoint assessment, 8 (32.0% [95% CI, 14.9%-53.5%]) of 25 evaluable participants had a clinically meaningful worsening in TUG time during the 24 weeks of treatment from baseline (inclusive of assessments at weeks 12 and 24).

**Figure 1.**
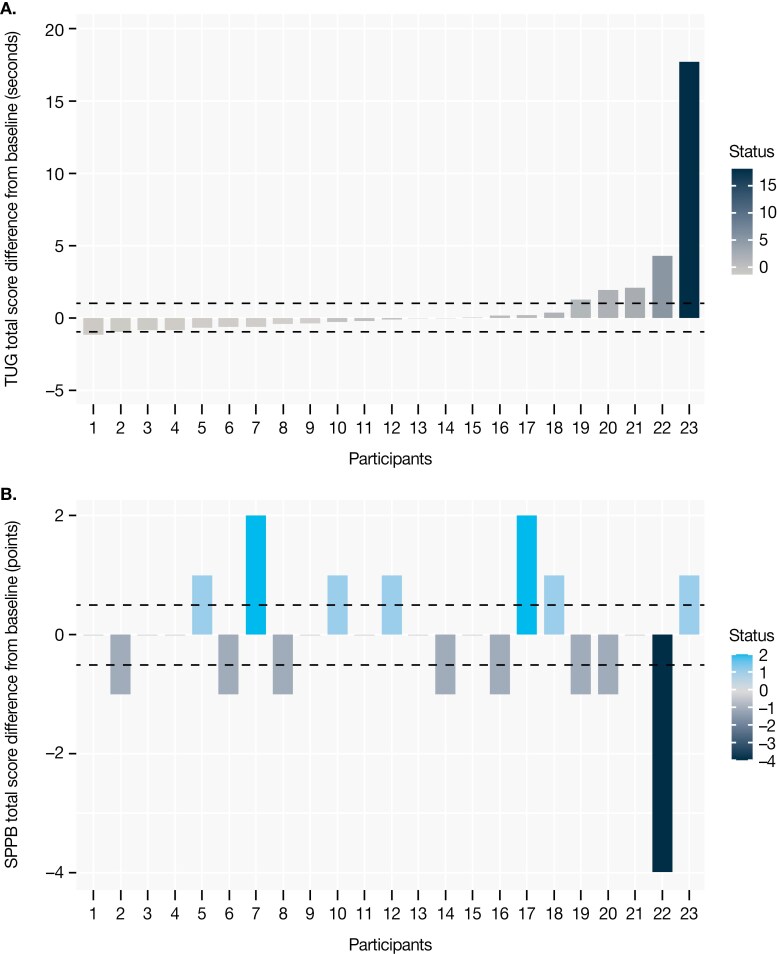
Proportion of participants^a^ at week 24 with a clinically meaningful worsening in (A) TUG time^b^ and (B) SPPB total score^c^ from baseline. ^a^Only participants with valid scores for both tests are displayed. Participants are displayed in the same order for both tasks. ^b^Worsening for the TUG test was defined as an increase of ≥1 second in TUG time from baseline. ^c^Worsening of the SPPB test was defined as a decline of ≥0.5 points in SPPB total score from baseline. Abbreviations: SPPB, Short Physical Performance Battery; TUG, Timed Up and Go.

A clinically meaningful worsening in TUG time specifically at the week 24 study visit was reported for 5 (21.7%) of 23 evaluable participants (Figure 1A), while a clinically meaningful worsening in total SPPB score at the week 24 study visit was reported for 8 (33.3%) of 24 evaluable participants (Figure 1B). There was no clear difference in the numbers of participants with worsening TUG time or SPPB scores at week 24 based on metastatic status or age subgroup (Figures S2 and S3). Among those evaluable at the week 52 visit, 5 (26.3%) of 19 participants had a clinically meaningful worsening in TUG time, and 7 (31.8%) of 22 participants had a clinically meaningful worsening in total SPPB score.

**Figure 2.**
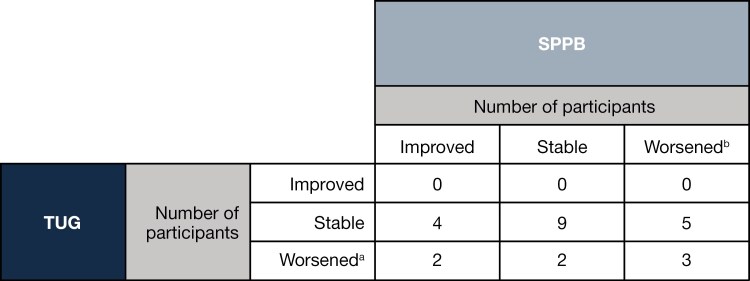
Test agreement during the 24 weeks from baseline. ^a^Worsening for the TUG test was defined as an increase of ≥1 second in TUG time from baseline. ^b^Worsening for the SPPB test was defined as a decline of ≥0.5 points in SPPB total score from baseline. Abbreviations: SPPB, Short Physical Performance Battery; TUG, Timed Up and Go.

There was poor agreement between results for the TUG and SPPB tests during the 24-week treatment period from baseline, with only 48% of individual participant results showing the same outcome, including 3 participants with a clinically meaningful worsening on both tests (Figure 2). Thirteen participants had incongruent results between the 2 tests (ie, clinically meaningful worsening in one test and improving or remaining stable in the other). Since the agreement of individual participant results between the TUG and SPPB tests in the lead-in phase was below the predefined threshold of ≥85%, the study was prematurely terminated prior to initiation of the randomized phase.

### Safety

Overall, 26 (86.7%) participants experienced ≥1 TEAE; events reported in ≥10% of participants included fatigue (*n* = 8 [26.7%]), hematuria (*n* = 5 [16.7%]), dizziness (*n* = 4 [13.3%]), maculopapular rash (*n* = 3 [10.0%]), back pain (*n* = 3 [10.0%]), pain in extremity (*n* = 3 [10.0%]), and coronavirus disease 2019 (COVID-19; *n* = 3 [10.0%]). Fourteen participants experienced ≥1 TEAE considered related to study treatment by the investigator; treatment-related TEAEs reported in ≥5% of participants included fatigue (*n* = 7 [23.3%]), dizziness (*n* = 3 [10.0%]), and maculopapular rash (*n* = 2 [6.7%]). Six (20.0%) participants experienced a grade ≥3 TEAE; the only grade ≥3 TEAE occurring in >1 participant was hypertension (*n* = 2 [6.7%]). One participant experienced grade ≥3 hypertension that was considered related to treatment by the investigator but resolved after 30 days. Two (6.7%) participants experienced adverse events of interest: one was a grade 2 fall that occurred 452 days after treatment initiation and the other was a spinal fracture occurring at 391 days after the first dose of study treatment; neither event was considered related to study treatment by the investigator. One participant died due to cardiac failure, which was not considered related to treatment.

## Discussion

In the lead-in phase of the DaroAcT study, approximately two-thirds of participants with CRPC showed no clinically meaningful worsening in physical functioning as measured using the TUG test during 24 weeks of treatment with darolutamide 600 mg twice daily. A similar proportion of participants showed no clinically meaningful worsening in physical functioning as measured using the SPPB test. Although the TUG and SPPB tests are not identical, both measure strength, walking speed, and stability and reflect the overall physical functioning of the patient.^27,28,36^ Because of this, a relatively high degree of agreement was expected between the 2 tests at an individual participant level. However, despite similar proportions of participants with worsening performance, there was poor agreement between the tests, with only 48% of individual participant results for TUG and SPPB showing mutually consistent outcomes. Some participants showed improvement in one test and worsening results in the other, which implied poor agreement. Given the infrequent agreement between the TUG and SPPB tests, results of the lead-in phase did not meet the criteria for initiating the subsequent randomized phase, and the study was thus terminated. Of note, analysis of the DaroAcT lead-in phase was limited by the small number of participants (*N* = 30), and greater agreement between tests may have been observed across a larger sample of patients with prostate cancer.

The lack of agreement between the TUG and SPPB tests seen in the current study deviates from what has been seen in previous studies on different clinical populations. In a cross-sectional study of patients with advanced knee osteoarthritis (*N* = 44), significant concurrent validity was observed between TUG and total SPPB scores.^50^ Similarly, in a nationwide multicenter study of community-dwelling elderly adults (*N* = 3010), a significant negative correlation between TUG time and total SPPB score was observed and remained after adjusting for sex and age.^51^ Finally, in an observational study of elderly adults in nursing homes (*N* = 45), scores obtained on the TUG and SPPB test were statistically correlated.^52^ Of note, however, individuals in these studies likely presented with impaired physical functioning at baseline. Thus, given that not all individuals with CRPC present with functional impairment, the lack of agreement seen in the current study may reflect a lack of sensitivity for these tests to detect minor changes in physical functioning in patients with CRPC specifically.

Several limitations associated with the TUG and SPPB tests may have contributed to the inconclusive results on physical functioning. First, the sample size of the DaroAct lead-in phase was small, and it is possible that greater agreement between tests may have been observed in a larger study population. Second, although both tests have been repeatedly validated in older adult populations, are frequently used to assess fall risk and frailty,^24-26,35,37,38^ and are recommended for measuring health-related quality of life by the Oncology EDGE Task Force for Prostate Cancer on Functional Mobility,^23^ neither test has been validated yet in patients with prostate cancer. The task force recommendations are instead based on experience in broader populations (eg, older adults) and characteristics such as ease of use. Although a decline in physical functioning with ADT use has been previously reported,^7-11,13,14^ it is possible that this decline is not severe enough to be reliably captured by the TUG and SPPB tests or that the decline takes longer to fully manifest itself than the study treatment period allowed. Additionally, while there have been limited reports of the TUG and SPPB tests being used to assess the effects of therapeutic interventions, mainly in primary motor or neuromotor diseases, such as Parkinson’s disease, multiple sclerosis, and sarcopenia,^53-55^ to our knowledge these tests have not been used to assess the impact of adverse effects of anticancer drugs on physical functioning. It is possible that the addition of ARI therapy in patients who have already experienced chronic androgen deprivation may have relatively modest effects on their physical impairment compared with the impact of initial ADT therapy. Conducting these evaluations in patients being treated in earlier disease settings, for instance when ARIs are combined with initial ADT for patients with metastatic castration-sensitive prostate cancer, could prove more sensitive.

Another limitation to the use of the TUG and SPPB tests is the substantial variability in the measurement procedures applied for these tests in the published literature. With respect to the TUG test, there are reported differences in the distance walked (2.5-10 m),^15,25^ walking pace (comfortable/usual walking speed or as fast as possible),^24,25,30,34,56^ and whether patients were allowed to practice^18,26,30,56^ or use an assistive device or support to aid their balance.^18,24^ Similarly, the methodology applied for the SPPB test has varied across studies, including different orders of the individual component test items,^22,37^ the threshold commonly used to define poor/abnormal performance,^57,58^ and the training of personnel administering the test. A recent study reported excellent test-retest reliability for the TUG test in older frail and non-frail patients with prostate cancer when administered on the same day by the same physiotherapist, suggesting the importance of controlling for methodologic variabilities.^59^ It should also be noted that, because TUG and SPPB are performance-based tests, there can be variability in the participants’ level of effort, even within an office visit/session. In the current study, all test assessors received training on standard operating procedures for both the TUG and SPPB tests. With multiple study sites and test assessors, however, inter-rater reliability (degree of agreement among independent observers or test assessors) is a potential limitation. While inter-rater reliability can be enhanced with training, inherent differences between assessors (ie, reaction time for stopping a stopwatch) may remain. Therefore, while assessments for each patient were performed according to standard operating procedures throughout the study, the small number of participants in DaroAcT, variation in patient effort, and potential inter-rater variability may have contributed to confounding results.

Despite the limitations discussed here and described in the literature, the TUG and SPPB tests remain important options for evaluating physical functioning in older adults. However, formal validation of these tests in future clinical trials in patients with prostate cancer is needed to provide greater clarity in regard to how physical functioning should be assessed in this specific patient population, particularly when related to the evaluation of potential short-term, treatment-related declines in physical functioning. It should be noted that other published studies have assessed physical functioning in patients with prostate cancer using alternate measures. One study assessed self-reported physical functioning in patients with metastatic prostate cancer receiving enzalutamide plus testosterone suppression or an active control (other hormonal treatment) and used the physical functioning domain of the European Organisation for Research and Treatment of Cancer core quality of life questionnaire.^14^ In this study, patients receiving enzalutamide reported an earlier and greater decline in physical functioning score compared with the active control, although it is unclear how sensitive self-reported tools are to early decrements in physical functioning. A separate study assessed physical functioning using individual measurements of grip strength, gait speed, and timed chair stands in patients with mCRPC aged ≥65 years who started treatment with docetaxel, abiraterone, enzalutamide, or radium-223.^13^ After 3 months of treatment, the radium-223 group had the highest proportion of patients with worsening in grip strength and gait speed, whereas the enzalutamide and docetaxel groups had the highest proportions of patients with worsening in chair stands and activities of daily living, respectively. Further work defining optimal methods and indications for assessing physical functioning in the advanced prostate cancer population is a critical component of understanding how to maximize patient outcomes over time and identify vulnerable patients who may benefit from additional supportive measures.

The safety findings in the current study are in agreement with previous safety reports for darolutamide in patients with prostate cancer.^7^ Notably, the 2 separate adverse events of fall and spinal fracture were considered unrelated to study treatment, which is relevant since other ARIs (enzalutamide and apalutamide) have been associated with an increased incidence of these adverse events.^8,9^

## Conclusions

In the lead-in phase of the phase 2b DaroAcT study, the proportion of participants achieving comparable scores in the TUG and SPPB tests during 24 weeks of treatment with darolutamide did not meet the criteria for study continuation, thus supporting the decision to terminate the study prior to initiating the randomized phase. While numerical changes in scores were observed, inferences should be made with caution due to the small sample size and the question as to whether both the TUG and SPPB tests reliably detect changes in physical function related to hormonal therapy specifically. When investigating the impact of prostate cancer interventions, it is important to assess physical functioning along with general efficacy and safety evaluations. Therefore, continued research is needed to help identify the optimal tool(s) that reliably and accurately captures changes in physical functioning in the prostate cancer setting.

## Supplementary material

Supplementary material is available at *The Oncologist* online.

oyae287_suppl_Supplementary_Figures_S1-S3

## Data Availability

Availability of the data underlying this publication will be determined according to Bayer’s commitment to the EFPIA/PhRMA “Principles for responsible clinical trial data sharing.” This pertains to scope, timepoint, and process of data access. As such, Bayer commits to sharing upon request from qualified scientific and medical researchers patient-level clinical trial data, study-level clinical trial data, and protocols from clinical trials in patients for medicines and indications approved in the United States (US) and European Union (EU), as necessary for conducting legitimate research. This applies to data on new medicines and indications that have been approved by the EU and US regulatory agencies on or after January 01, 2014. Interested researchers can use www.vivli.org to request access to anonymized patient-level data and supporting documents from clinical studies to conduct further research that can help advance medical science or improve patient care. Information on the Bayer criteria for listing studies and other relevant information is provided in the member section of the portal. Data access will be granted to anonymized patient-level data, protocols, and clinical study reports after approval by an independent scientific review panel. Bayer is not involved in the decisions made by the independent review panel. Bayer will take all necessary measures to ensure that patient privacy is safeguarded.
