# Supplementary material for: Functional assessment in patients with castration-resistant prostate cancer treated with darolutamide: results from the DaroAcT study
Source: Oncologist. 2024 Oct 25;30(7):oyae287. doi: 10.1093/oncolo/oyae287 (PMC12311285; doi:10.1093/oncolo/oyae287)

**Supplementary Appendix**

**Supplementary Figure S1. Study Design*.*** ADT, androgen deprivation therapy; CRPC, castration-resistant prostate cancer. ^a^The study planned to enroll 150 participants in total; 30 in lead-in phase and 120 in randomized phase. ^b^All participants continued to receive ADT of the investigator's choice (luteinizing hormone-releasing hormone agonist/antagonists) as standard therapy or were required to have had an orchiectomy.


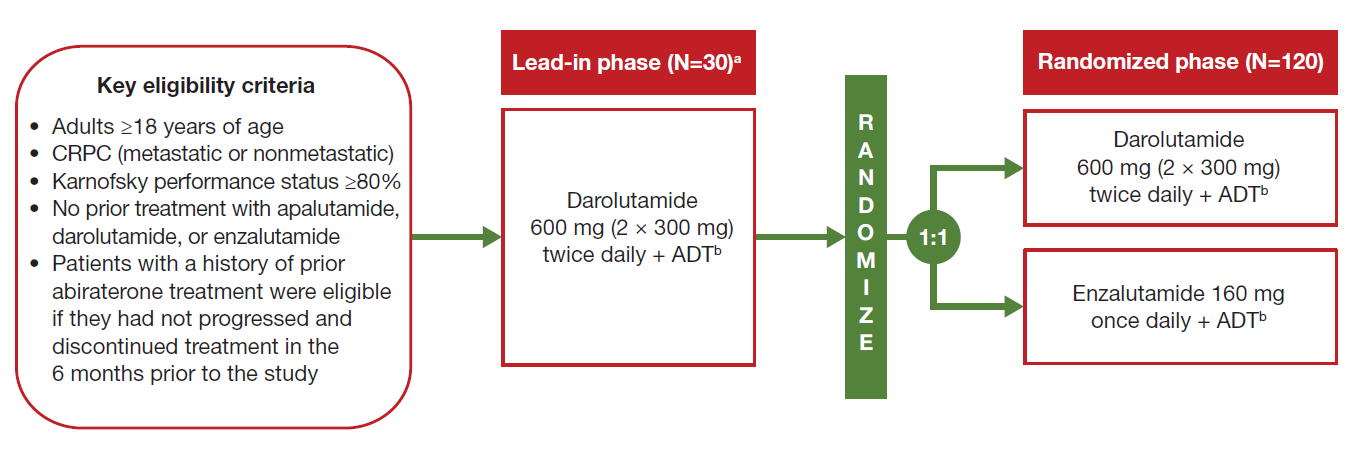


**Supplementary Figure S2. Proportion of Participants at Week 24 With a Clinically Meaningful Worsening in TUG Time*^a^* From Baseline by Metastatic Status and Age.** TUG, Timed Up and Go. ^a^Worsening for the TUG test was defined as an increase of ≥1 second in TUG time from baseline.


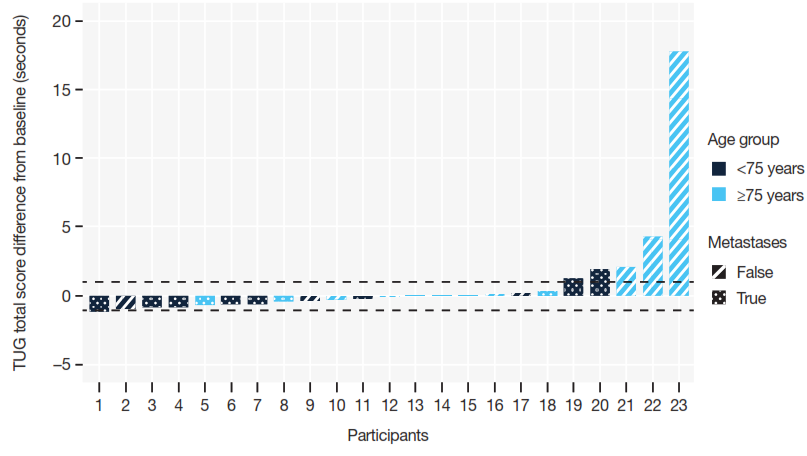


**Supplementary Figure S3. Proportion of Participants at Week 24 With a Clinically Meaningful Worsening in SPPB Total Score*^a^* From Baseline by Metastatic Status and Age*.*** SPPB, Short Physical Performance Battery. ^a^Worsening for the SPPB test was defined as a decline of ≥0.5 points in SPPB total score from baseline.


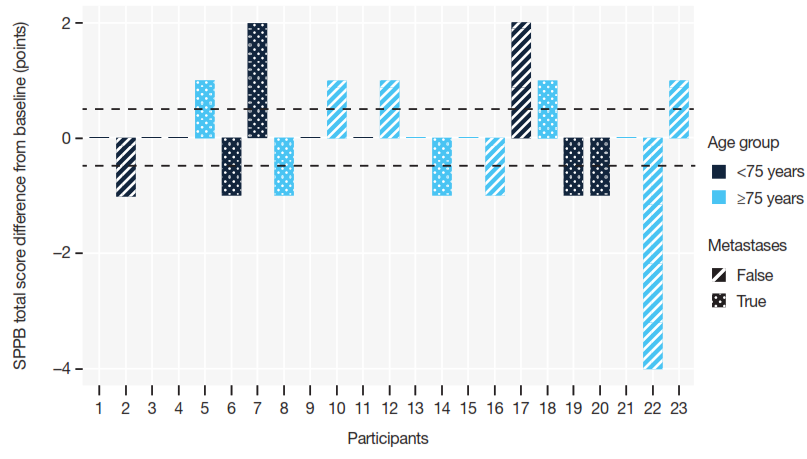

Supplement: oyae287_suppl_Supplementary_Figures_S1-S3 [file oyae287_suppl_supplementary_figures_s1-s3.docx]
